# Automated substance testing for lab-on-chip devices

**DOI:** 10.1186/1753-6561-7-S6-P28

**Published:** 2013-12-04

**Authors:** Lutz Kloke, Katharina Schimek, Sven Brincker, Alexandra Lorenz, Annika Jänicke, Christopher Drewell, Silke Hoffmann, Mathias Busek, Frank Sonntag, Norbert Danz, Christoph Polk, Florian Schmieder, Alexey Borchanikov, Viacheslav Artyushenko, Frank Baudisch, Mario Bürger, Reyk Horland, Roland Lauster, Uwe Marx

**Affiliations:** 1 Technische Universität Berlin/Germany; 2 Fraunhofer IWS, Dresden/Germany; 3 GeSiM mbh, Großerkmannsdorf/Germany; 4 ART Photonics GmbH, Berlin/Germany

## Background

A smartphone-sized multi-organ-chip has been developed by TissUse. This platform consists of a microcirculation system which contains several fully endothelial-cell-coated micro- channels in which organ equivalents are embedded. Briefly, Human 3D organ equivalents such as liver and skin could be maintained functional over 28 days and treated with chemical entities in this microcirculation system.

In order to automate the Multi-Organ-Chip (MOC) handling we developed with partners a robotic platform. The prototype is capable to maintain 10 MOCs. Operations can be programmed individually by its user. For example OECD guidelines for acute toxicity testing could be performed. The robotic platform features also functions such as automatic media supply, sampling and storage, temperature control, fluorescence and microscopic monitoring, PIV, O2-measurement, etc. To display the functionality we performed a toxicity test with RPTEC cells treated with DMSO in different concentrations.

## Proof of concept study

RPTEC cells were used as cellular model system. The cells were cultivated in two Generation-4-MOCs as well as in 96-well-plates working as reference system. The systems were stained with CellTracker™ Red and cultivated at 37°C and 5% CO_2 _saturation. After some hours of resting MOCs and MWPs were treated with 10% respectively 20% DMSO. Afterwards the fluorescence activity was measured in 20 minute intervals in order to detect potential cell death. The cells can be detected by the monitoring unit of the robot. A 20 μmol/L CellTracker™ Red staining provides a sufficient signal which can be monitored over time. The treatment with 10% DMSO shows a fluorescence signal decline of more than 50% and the following recovery of them.

## Summary

This project shows the successful development of a robotic platform to handle multi-organ-chips. Maintenance as well as user specific protocols, for example toxicity testing, can be accomplished with a minimum amount of labor time. The MOCs in combination with the robotic platform offer the plug-and-play solution to generate substance interaction data on a Lab-on-Chip system.

